# Surgical explantation of the WATCHMAN device and right atrial thrombus: a case report

**DOI:** 10.1093/jscr/rjae601

**Published:** 2024-09-23

**Authors:** Kotaro Mukasa, Hironobu Nishiori, Hiroki Ikeuchi, Tomohiko Inui, Goro Matsumiya

**Affiliations:** Department of Cardiovascular Surgery, Chiba University Hospital, Chiba 286-0041, Japan; Department of Cardiovascular Surgery, Chiba University Hospital, Chiba 286-0041, Japan; Department of Cardiovascular Surgery, Chiba University Hospital, Chiba 286-0041, Japan; Department of Cardiovascular Surgery, Chiba University Hospital, Chiba 286-0041, Japan; Department of Cardiovascular Surgery, Chiba University Hospital, Chiba 286-0041, Japan

**Keywords:** WATCHMAN, left atrial appendage closure, peri-device leakage, right atrial thrombus

## Abstract

A 74-year-old male with a history of cardioembolic stroke, chronic atrial fibrillation, and cerebral hemorrhage, who had undergone left atrial appendage closure using the WATCHMAN device 1 year prior, was diagnosed with a 25-mm intra-cardiac mass in the right atrium. The patient underwent the surgical removal of the right atrial mass and the explantation of the WATCHMAN device. The WATCHMAN device was explanted with an external incision at the base of the left atrial appendage, facilitating the removal of the device and the closure of the appendage through direct suturing. The pathological examination confirmed the right atrial mass to be a thrombus. The patient was discharged on postoperative Day 13. From the perspectives of simplicity and radicality, the external approach could be a good option.

## Introduction

With the increasing cases of percutaneous left atrial appendage (LAA) close using a WATCHMAN device (Boston Scientific, Marlborough, MA), the number of cases requiring the extraction of the device will also increase. In cases of device-related thrombus, continued anticoagulation therapy is generally deemed adequate. However, it often becomes inevitable to stop anticoagulation and explant the WATCHMAN device. Several techniques are reported; explantation with primary closure of LAA through a trans-left atrial approach, or exclusion with an AtriClip (AtriCure, Mason, Ohio) if anatomically suitable [1]. We report a case that, 1 year after WATCHMAN implantation, a right atrial thrombus was formed, requiring simultaneous explantation of the thrombus and the WATCHMAN device due to peri-device leak. The procedure involved an external approach for LAA resection and device removal, followed by direct suturing of the LAA, resulting in safe and successful removal.

## Case report

**Figure 1:**
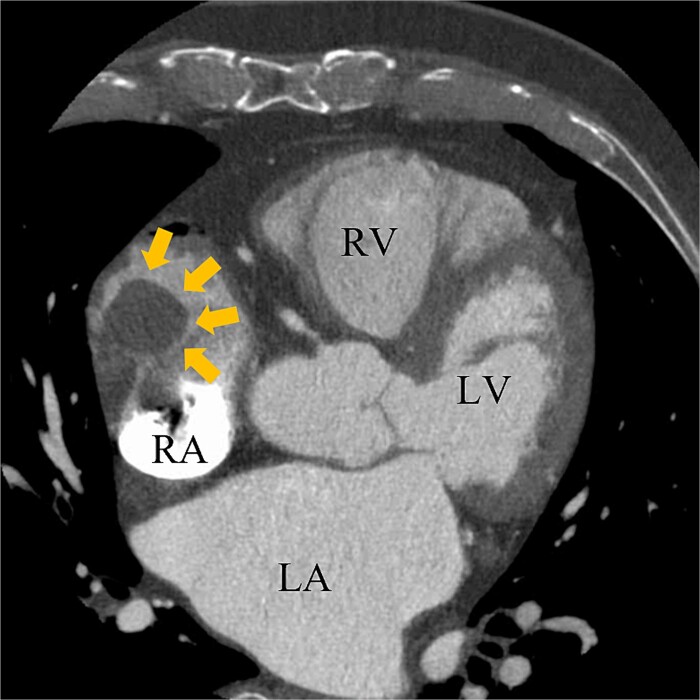
Preoperative enhanced computed tomography imaging showing a 25-mm intra-cardiac mass in the RA (arrows). RV, right ventricle; LA, left atrium; LV, left ventricle.

**Figure 2:**
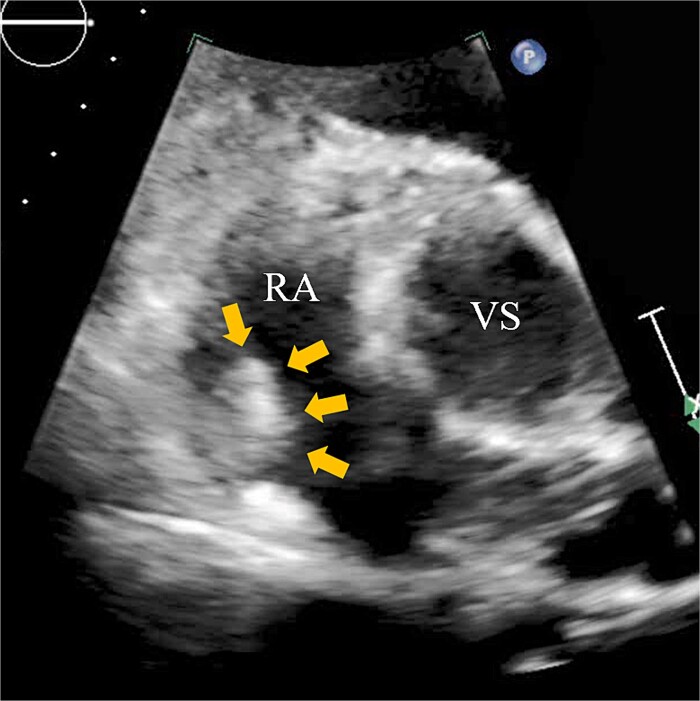
Transthoracic echocardiography showing a right atrial mass measuring 17 × 19 mm with slight mobility (arrows). VS, Valsalva sinus.

**Figure 3:**
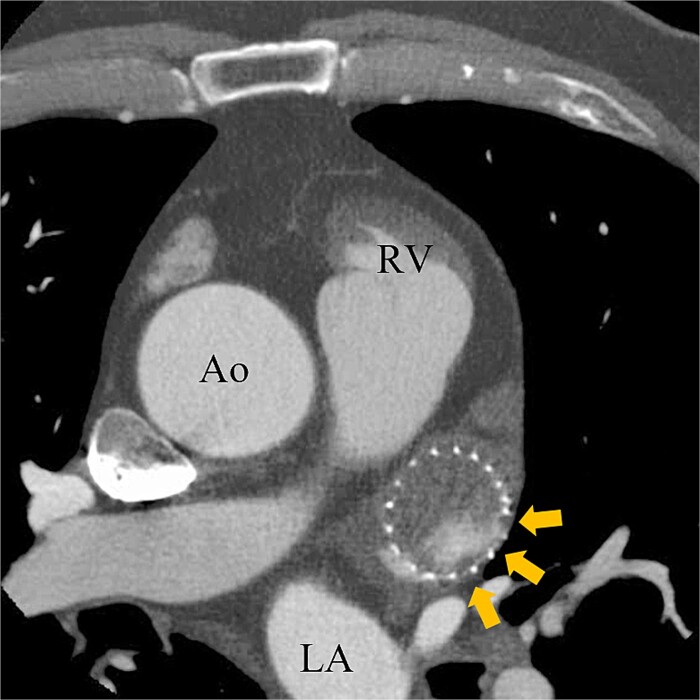
Preoperative enhanced computed tomography imaging showing peri-device leakage around the WATCHMAN (arrows). Ao, aorta.

**Figure 4:**
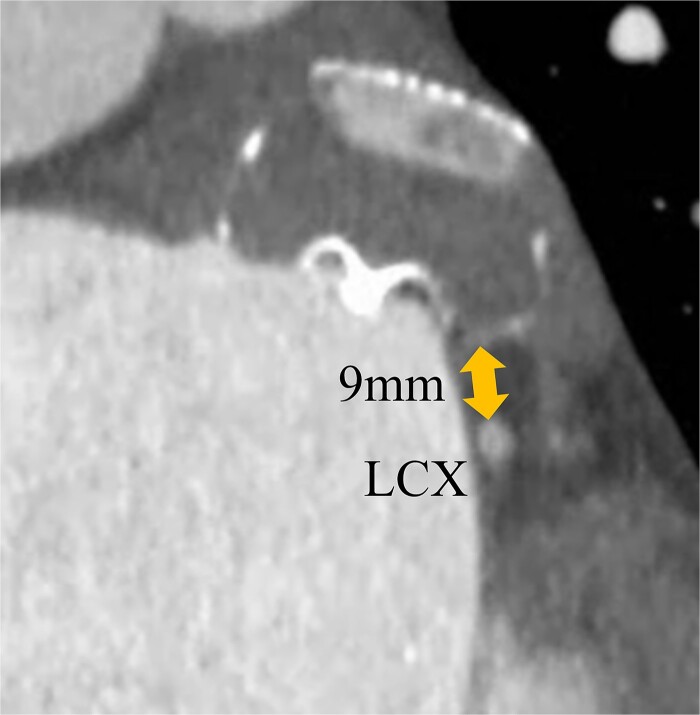
Preoperative enhanced computed tomography imaging showing the distance between the WATCHMAN device and the LCX was ~9 mm.

A 74-year-old male with a history of chronic atrial fibrillation (AF) for over 10 years, cardioembolic stroke, and cerebral hemorrhage, who had undergone LAA closure using the WATCHMAN device 1 year prior, was admitted after a follow-up contrast-enhanced CT scan revealed a 25-mm intracardiac mass in the right atrium (RA) (Fig. 1). After the WATCHMAN implantation, the patient was initially on direct oral anticoagulant (DOAC) but switched to dual antiplatelet therapy (DAPT) following the placement of a drug-eluting stent during percutaneous coronary intervention performed 9 months later. Transthoracic echocardiography revealed a left ventricular ejection fraction of 61% and a right atrial mass measuring 17 × 19 mm with slight mobility (Fig. 2). Despite anticoagulation therapy with heparin, the right atrial mass did not reduce in size. The patient decided to undertake surgical removal of the mass due to the potential risks of tumor or embolization. Additionally, contrast-enhanced CT showed a peri-device leak around the WATCHMAN device (Fig. 3). Considering the patient’s history of cerebral hemorrhage and the anticipated future cessation of anticoagulation therapy, we determined to perform WATCHMAN device explantation and LAA closure. Preoperative contrast-enhanced CT showed no thrombi attached to the WATCHMAN device and sufficient distance from the left circumflex artery (LCX) and the device (Fig. 4). We planned to remove the device by incising from the outside surface of the LAA. The Maze procedure was not performed because it was considered less effective due to the patient’s history of AF lasting more than 20 years and the presence of flat f-waves in lead V1 on the 12-lead electrocardiogram.

**Figure 5:**
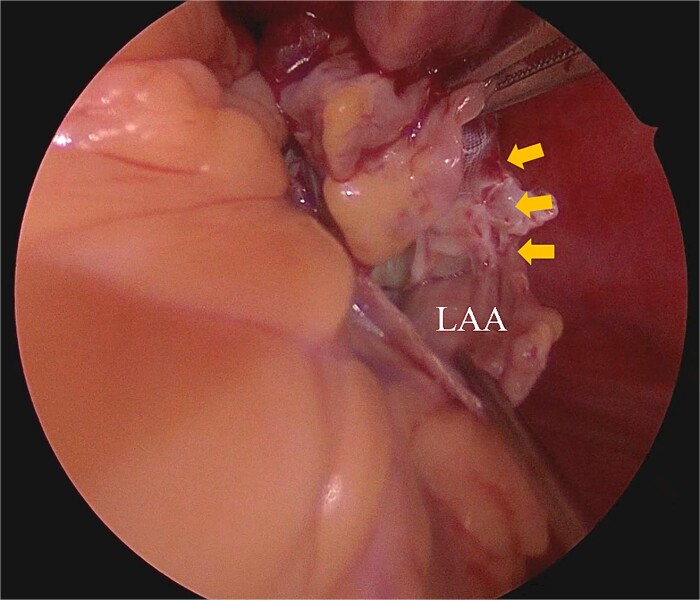
Intraoperative finding shows that the piece of WATCHMAN device can be shown from an external incision of LAA.

Under general anesthesia, the chest was entered through a median sternotomy. Cardiopulmonary bypass was established with dual venous drainage. The aorta was cross-clamped, and cardiac arrest was achieved through antegrade cardioplegia. Upon right atriotomy, the right atrial mass was attached to the ventral wall of the RA; it was easily excised by scissors. Subsequently, palpation of the LAA confirmed that the WATCHMAN device was adequately deep-seated and there was enough room from the base of the LAA. An incision was made at the base of the LAA, and the WATCHMAN device was excised along with the LAA (Fig. 5). The LAA was closed with direct suturing. The pathological examination confirmed the right atrial mass to be a thrombus. Postoperatively, anticoagulation therapy with warfarin was initiated. The patient was discharged from the hospital 13 days postsurgery. The echocardiogram performed 6 months postoperatively that did not reveal any recurrence of mass.

## Discussion

The surgical approach in this case involved creating an external incision of the LAA, enabling the extraction of the WATCHMAN device with the LAA followed by direct suturing. Sarah T. and her colleagues have reported cases necessitating surgical interventions via the trans-LA approach, which included direct extraction of the device followed by direct closure or the application of a bovine pericardial patch for the LAA orifice [1]. These approaches could avoid damage to surrounding structures during the detachment and extraction of the WATCHMAN device.

However, primary endocardial suture closure of the LAA has a re-opening risk rate of 36% [2]. There might be a similar risk of re-opening with the exclusion of the LAA using a bovine pericardial patch. Consequently, we have adopted the external approach that involves removing the LAA along with the WATCHMAN device. This technique offers the advantages of being performed without an extra incision at the right side of the left atrium, enabling the entire resection of the LAA, and the risk of thrombus formation is reduced significantly. However, in cases where thrombi are adherent to the device, there is a risk of thrombus dislodging into the cardiac chamber. When the device is close to the LCX, there is a risk of arterial injury. Under these circumstances, this approach is inappropriate. Surgeons are required to evaluate the position, size, and thrombus to select appropriate methods.

The emergence of a right atrial thrombus is uncommon. Right atrial thrombi are detected in 0.03% of patients with AF [3]. Considering Virchow’s triad, the conditions conducive to the formation of right atrial thrombi can be categorized into issues with the endocardium, blood flow, and blood coagulability. Specific examples include endocardial damage due to cardiomyopathy, indwelling central venous catheters or pacemaker leads, AF, and congenital thrombophilic conditions [4–6]. AF is considered a contributing factor in this case. Furthermore, there are reports of right atrial thrombi following percutaneous transvenous mitral commissurotomy, suggesting that endocardial damage from wire manipulation during the WATCHMAN implantation procedure and the involvement of Brockenbrough’s needle could also be a factor [7]. Considering the thrombi emerged after switching from DOAC to DAPT, it is possible that endothelial damage existed, and it became manifest upon cessation of anticoagulation therapy.

We report a case where right atrial thrombectomy and removal of the WATCHMAN device were conducted. Under permissible conditions, the external approach for the WATCHMAN explantation may serve as an efficacious option.

## Data Availability

Not applicable.
